# Differential Expression and Localization of ADAMTS Proteinases in Proliferative Diabetic Retinopathy

**DOI:** 10.3390/molecules27185977

**Published:** 2022-09-14

**Authors:** Ahmed M. Abu El-Asrar, Mohd Imtiaz Nawaz, Eef Allegaert, Mohammad Mairaj Siddiquei, Ajmal Ahmad, Priscilla Gikandi, Gert De Hertogh, Ghislain Opdenakker

**Affiliations:** 1 Department of Ophthalmology, College of Medicine, King Saud University, PO Box 245, Riyadh 11411, Saudi Arabia; 2 Dr. Nasser Al-Rashid Research Chair in Ophthalmology, College of Medicine, King Saud University, PO Box 245, Riyadh 11411, Saudi Arabia; 3 Laboratory of Histochemistry and Cytochemistry, University of Leuven, KU Leuven, 3000 Leuven, Belgium and University Hospitals UZ Gasthuisberg, 3000 Leuven, Belgium; 4 Rega Institute for Medical Research, Department of Microbiology and Immunology and Transplantation, University of Leuven, KU Leuven, 3000 Leuven, Belgium and University Hospitals UZ Gasthuisberg, 3000 Leuven, Belgium

**Keywords:** proliferative diabetic retinopathy, ADAMTS proteinases, MMP-15, versican, biglycan

## Abstract

We analyzed the expression of ADAMTS proteinases ADAMTS-1, -2, -4, -5 and -13; their activating enzyme MMP-15; and the degradation products of proteoglycan substrates versican and biglycan in an ocular microenvironment of proliferative diabetic retinopathy (PDR) patients. Vitreous samples from PDR and nondiabetic patients, epiretinal fibrovascular membranes from PDR patients, rat retinas, retinal Müller glial cells and human retinal microvascular endothelial cells (HRMECs) were studied. The levels of ADAMTS proteinases and MMP-15 were increased in the vitreous from PDR patients. Both full-length and cleaved activation/degradation fragments of ADAMTS proteinases were identified. The amounts of versican and biglycan cleavage products were increased in vitreous from PDR patients. ADAMTS proteinases and MMP-15 were localized in endothelial cells, monocytes/macrophages and myofibroblasts in PDR membranes, and ADAMTS-4 was expressed in the highest number of stromal cells. The angiogenic activity of PDR membranes correlated significantly with levels of ADAMTS-1 and -4 cellular expression. ADAMTS proteinases and MMP-15 were expressed in rat retinas. ADAMTS-1 and -5 and MMP-15 levels were increased in diabetic rat retinas. HRMECs and Müller cells constitutively expressed ADAMTS proteinases but not MMP-15. The inhibition of NF-κB significantly attenuated the TNF-α-and-VEGF-induced upregulation of ADAMTS-1 and -4 in a culture medium of HRMECs and Müller cells. In conclusion, ADAMTS proteinases, MMP-15 and versican and biglycan cleavage products were increased in the ocular microenvironment of patients with PDR.

## 1. Introduction

The development and progression of proliferative diabetic retinopathy (PDR) is a complex process that requires the temporal and spatial coordination of different events including inflammation, ischemia-induced retinal angiogenesis and fibrosis. These processes are regulated by growth factors, proinflammatory cytokines, chemokines, adhesion molecules and proteolytic enzymes. The outgrowth of fibrovascular epiretinal membranes at the vitreoretinal interface is the pathological hallmark feature of PDR. The outgrowth of fibrovascular tissue often leads to serious vision loss due to recurrent vitreous hemorrhage and/or traction retinal detachment. The retinal microvasculature is progressively damaged in diabetic patients, resulting in various events such as retinal ischemia, the upregulation of hypoxia inducible factor-1, and vascular endothelial growth factor (VEGF) secretion, possibly progressing to PDR, which is diagnosed according to the presence of vascular lesions [1,2,3,4,5,6,7,8]. The remodeling and degradation of the extracellular matrix (ECM) are key factors in the initiation and progression of angiogenesis [9]. In the ocular microenvironment of patients with PDR, several matrix metalloproteinases (MMPs) are upregulated, and these MMPs are thought to play an active role in promoting the neovascular process [1,2,3,4]. Aside from the MMP family with about 20 proteinases in the human species, the superfamily of ADAMTS (a disintegrin and metalloproteinase with thrombospondin motifs) contains members belonging to the catalytic class of zinc-containing metalloproteinases and members that lack catalytic activity (ADAMTS-like proteins) [10]. All ADAMTS superfamily members are secreted glycoproteins, and the subfamily of ADAMTS proteinases mediate diverse functions, including ECM degradation during angiogenesis, inflammation and tumor growth [11,12]. In addition to the catalytic domain, located in the aminoterminal part, and various ancillary domains positioned carboxyterminally, ADAMTS proteinases contain a variable number of thrombospondin-1-like repeats (TSRs) in their ancillary region. The TSRs are shown to regulate angiogenesis by modulating VEGF activity [12].

The human ADAMTS proteinase family can be functionally subgrouped on the basis of their known substrates. For instance, proteoglycan degradation may be executed by ADAMTS-1, ADAMTS-4, ADAMTS-5, ADAMTS-8, ADAMTS-9, ADAMTS-15 and ADAMTS-20, whereas aminoterminal pro-collagen processing is by ADAMTS-2, ADAMTS-3 and ADAMTS-14, and von Willebrand factor (vWF) is cleaved by ADAMTS-13 [11,12]. The proteolytic cleavage of the proteoglycan substrates versican and biglycan is implicated in angiogenesis. Several studies established the enzyme/substrate relationship between these two groups of molecules in the setting of vascular dysfunction and angiogenesis [13,14,15].

Little attention has been focused on the expression and regulation of members of the ADAMTS proteinase family in the ocular microenvironment of patients with PDR. Because inflammation, angiogenesis and fibrosis are hallmarks of PDR, we analyzed the expression of the ADAMTS family members ADAMTS-1, -2, -4, -5 and -13 and the degradation products of the proteoglycan substrates versican and biglycan in the ocular microenvironment of patients with PDR. In addition, we investigated the effect of various diabetic-retinopathy-associated mechanisms on the expression of ADAMTS proteinases in retinal Müller glial cells and human retinal microvascular endothelial cells (HRMECs). The behavior of the ADAMTS-family members responsible for the cleavage of the proteoglycans of the interstitial components in the retina of diabetic patients has not been studied so far.

## 2. Results

### 2.1. Western Blot Analysis of Vitreous Samples from Patients with PDR and Nondiabetic Control Patients

**Figure 1:**
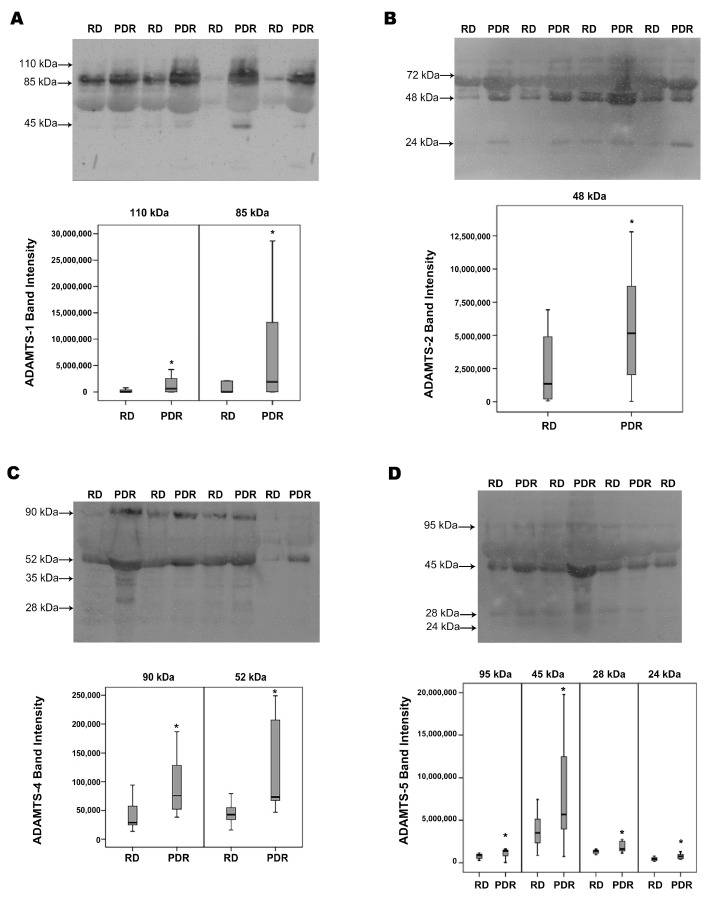

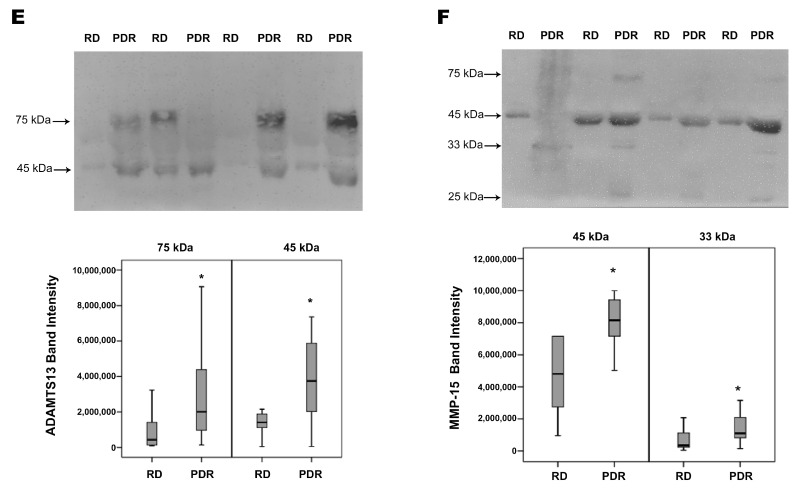
Determination of ADAMTS-1 (**A**), -2 (**B**), -4 (**C**), -5 (**D**) and -13 (**E**) and MMP-15 (**F**) levels in vitreous fluid samples. Equal volumes (15 µL) of vitreous fluid samples from patients with proliferative diabetic retinopathy (PDR; *n* = 16) and from nondiabetic patients with rhegmatogenous retinal detachment (RD; *n* = 16) were subjected to gel electrophoresis, and the presence of ADAMTS proteinases and MMP-15 were detected by Western blot analysis. Representative sets of samples are shown. The intensity of the protein bands was determined in all samples. Band intensities were compared between RD and PDR groups. Results are expressed as a median (interquartile range). (* *p* < 0.05; Mann–Whitney test).

With the use of Western blot analysis of equal volumes of vitreous fluid, we demonstrated the presence of ADAMTS-1, -2, -4, -5 and -13 in vitreous samples. ADAMTS-1 immunoreactivities were expressed as three protein bands at approximately 110 kDa, 85 kDa and 45 kDa. These corresponded to the proform (zymogen form) at around 110 kDa and the further processed active/mature forms at around 85 kDa and 45 kDa [16]. As shown in Figure 1A, the predominant ADAMTS-1 proteoform was found at 85 kDa indicating that ADAMTS-1 in vitreous samples was largely the mature form. A scanning analysis of immunoreactivities indicated increased levels of the proform 110 kDa and mature 85 kDa ADAMTS-1 in PDR versus control patient vitreous samples.

ADAMTS-2 immunoreactivities were expressed as three protein bands at approximately 72 kDa, 48 kDa and 24 kDa. The predominant ADAMTS-2 observed in vitreous fluid was around 48 kDa (Figure 1B), and the levels of this proteoform were also increased in vitreous fluids from PDR patients in comparison with those from control subjects. The lower-molecular-weight bands of ADAMTS-2 might be attributed to the post-secretory processing of the full-length form.

ADAMTS-4 immunoreactivities were expressed as four protein bands at 90 kDa, 52 kDa, 35 kDa and 28 kDa (Figure 1C). The full-length proform human ADAMTS-4 (zymogen form) has been described to be 90 kDa. The predominant ADAMTS-4 observed in vitreous fluid was around 52 kDa, suggesting that the prodomain is cleaved efficiently to produce the mature/processed form prior to secretion into the vitreous fluid [17,18,19,20]. With the use of scanning analysis, we were able to document increased levels of ADAMTS-4 in the vitreous of PDR patients in comparison with controls for the 90 kDa proform and for the mature 52 kDa form.

ADAMTS-5 immunoreactivities were expressed as four protein bands at approximately 95 kDa, 45 kDa, 28 kDa and 24 kDa (Figure 1D). Whereas the 95kDa and 45 kDa proteoforms represent the proform and the major activation form, respectively, the minor immunoreactivities at lower molecular masses might represent different degradation fragments [21]. Most of the ADAMTS-5 immunoreactivity appeared at the level of 45 kDa form, as analyzed by densitometry. Although the levels of all of the immunoreactive ADAMTS-5 forms were higher in PDR vitreous than in control samples, the difference was most prominent for the mature 45 kDa proteoform, which may result from higher expression levels in combination with the better processing of the proform because of the increased levels of processing enzymes in PDR.

ADAMTS-13 immunoreactivities were expressed as two protein bands. The approximate molecular weights of these bands were around 75 kDa and 45 kDa (Figure 1E). These forms represented truncated forms of the intact 190 kDa ADAMTS-13 [12,22], and the levels of both proteoforms were significantly increased in PDR vitreous when compared to control samples.

MMP-15 immunoreactivities were expressed as four protein bands at approximately 75 kDa, 45 kDa, 33 kDa and 25 kDa. Most of the MMP-15 immunoreactivity appeared at the level of the 45 kDa form (Figure 1F). In analogy with the increases in the densitometric band intensities of the expression of the studied ADAMTS proteinases in samples from PDR patients compared to samples from nondiabetic control patients, the levels of MMP-15 were significantly increased in PDR-patient-derived samples.

### 2.2. Increased Degradation Products of Versican and Biglycan in Vitreous Samples from Patients with PDR

**Figure 2:**
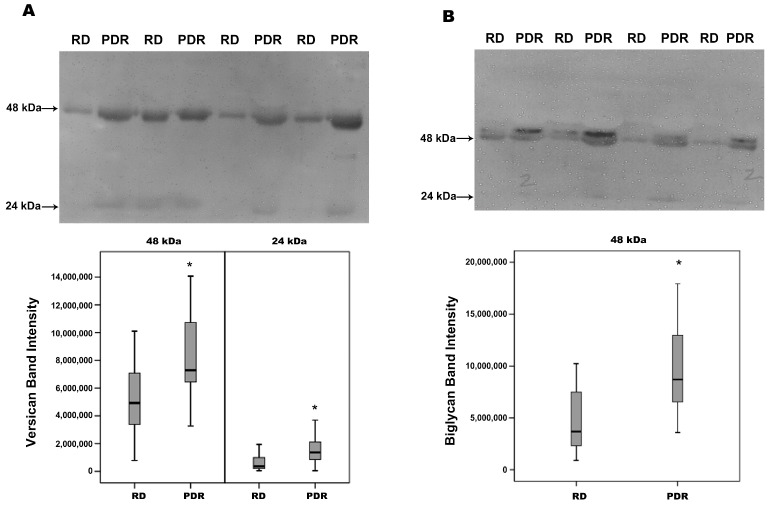
Determination of versican (**A**) and biglycan (**B**) degradation products in vitreous fluid samples. Equal volumes (15 µL) of vitreous fluid samples from patients with proliferative diabetic retinopathy (PDR; *n* = 16) and from nondiabetic patients with rhegmatogenous retinal detachment (RD; *n* = 16) were subjected to gel electrophoresis, and the presence of versican and biglycan degradation products was detected by Western blot analysis. Representative sets of samples are shown. The intensity of the protein bands was determined in all samples. Band intensities were compared between the RD and PDR groups. Results are expressed as a median (interquartile range). (* *p* < 0.05; Mann–Whitney test).

With the use of Western blot analysis, we showed that versican proteoforms were present at 48 kDa and 24 kDa. As shown in Figure 2A, the prominent versican band was found at 48 kDa. Biglycan antigens were also detected at 48 kDa and 24 kDa. The prominent biglycan band was found at 48 kDa (Figure 2B). Densitometric analysis of the bands revealed a significant increase in the levels of the degradation products of versican and biglycan in vitreous samples from patients with PDR compared to samples from nondiabetic control patients.

### 2.3. Neovascularization and Expression of the Monocyte/Macrophage Marker CD68 and the Myofibroblast Marker α-SMA in Epiretinal Fibrovascular Membranes from Patients with PDR

**Figure 3:**
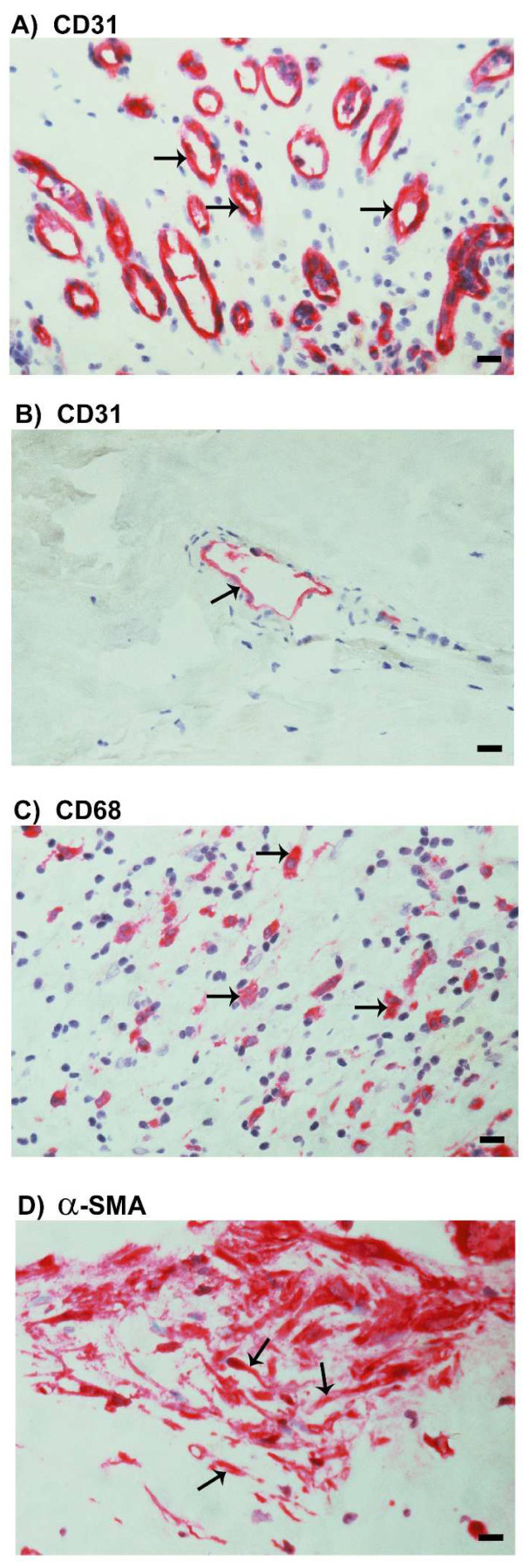
Immunohistochemical staining of epiretinal fibrovascular membranes from patients with proliferative diabetic retinopathy (PDR). Immunohistochemical staining for the endothelial cell marker CD31 showing pathologic new blood vessels expressing CD31 in a membrane from a patient with active neovascularization (arrows) (**A**) and in a membrane from another patient with involuted PDR, which is composed mostly of fibrous tissue (arrow) (**B**). Immunohistochemical staining for CD68 showing infiltrating monocytes/macrophages and neutrophil granulocytes in the stroma (arrows) (**C**). Immunohistochemical staining for α-smooth muscle actin (α-SMA) showing immunoreactivity in mainly spindle-shaped myofibroblasts (**D**) (scale bar, 10 µm).

As a negative control, the immunohistochemical staining procedure was performed with omission of the primary antibody from the protocol. No staining was observed in the negative control slides. Staining for the vascular endothelial cell marker CD31 was performed to determine the levels of angiogenic activity in epiretinal fibrovascular membranes from patients with PDR. All membranes showed neovessels that were positive for CD31. Representative stainings for CD31 in active (Figure 3A) and involuted (Figure 3B) membranes are shown. Monocytes/macrophages, neutrophil granulocytes expressing CD68 (Figure 3C) and spindle-shaped myofibroblasts expressing α-SMA (Figure 3D) were detected in all membranes.

### 2.4. Expression of ADAMTS Proteinases in Epiretinal Fibrovascular Membranes from Patients with PDR

**Figure 4:**
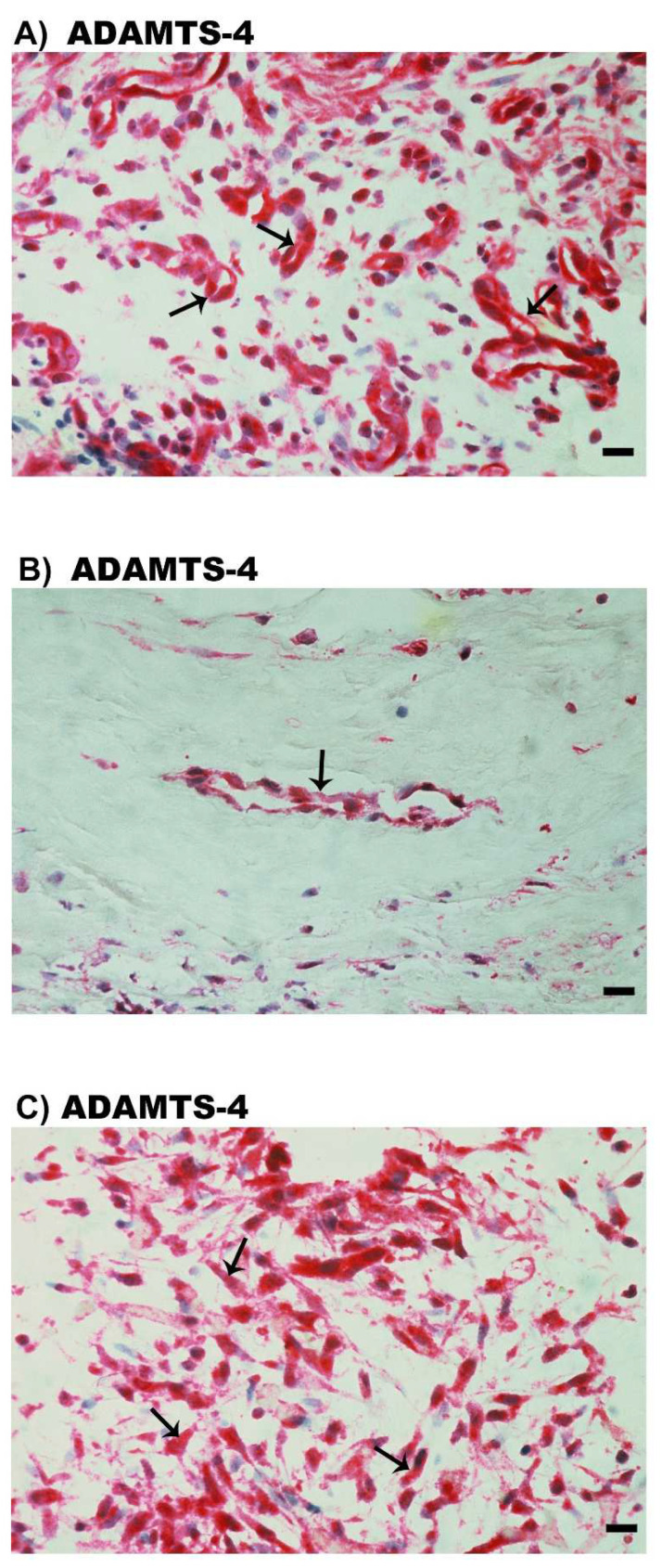
Immunohistochemical staining of epiretinal fibrovascular membranes from patients with proliferative diabetic retinopathy (PDR). Immunohistochemical staining for ADAMTS-4 showing immunoreactivity in vascular endothelial cells in a membrane from a patient with active neovascularization (arrows) (**A**) and in a membrane from a patient with involuted PDR (arrow) (**B**). Immunoreacivity for ADAMTS-4 was also detected in stromal spindle-shaped myofibroblasts (arrows) (**C**) (scale bar, 10 µm).

**Figure 5:**
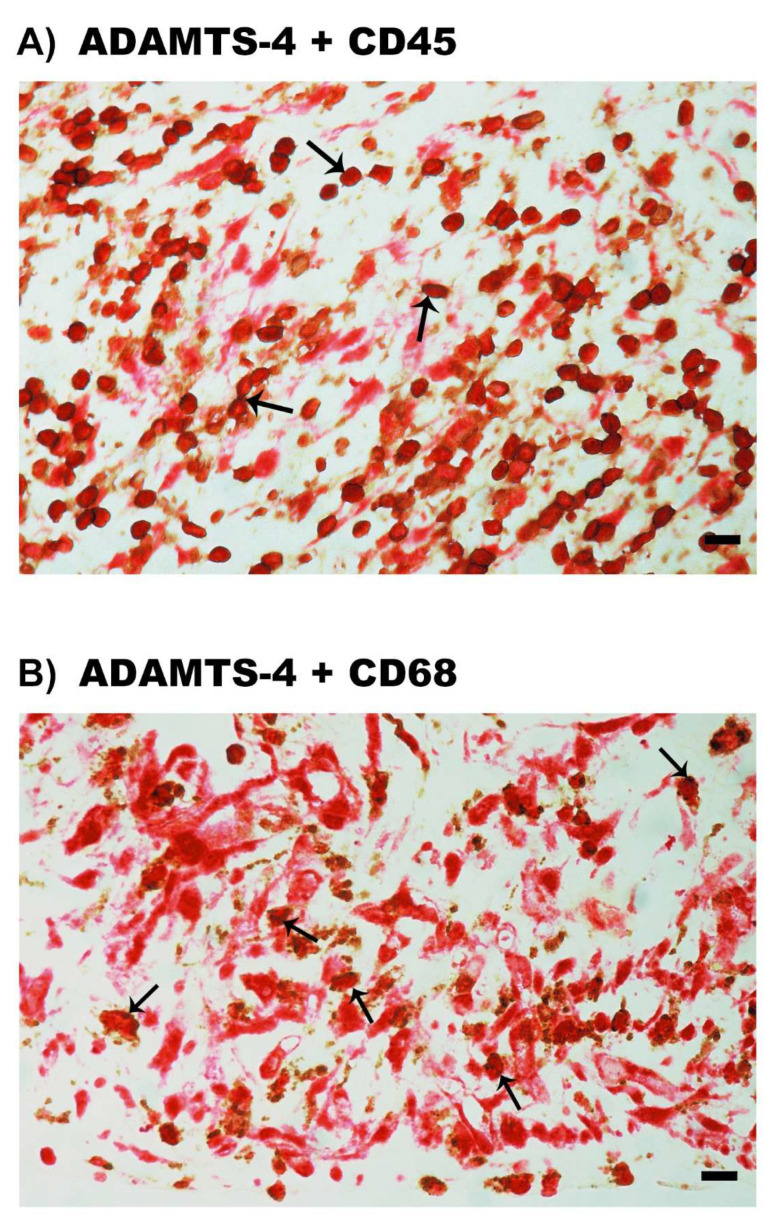
Immunohistochemical staining of epiretinal fibrovascular membranes from patients with proliferative diabetic retinopathy. Double immunohistochemical staining for ADAMTS-4 (red) and CD45 (brown) (**A**) or CD68 (brown) (**B**), showing co-expression in stromal cells. No counterstain to visualize the cell nuclei was applied (arrows) (scale bar, 10 µm).

We next wanted to identify the local cellular source of vitreous fluid ADAMTS proteinases and to examine the tissue expression and localization of ADAMTS proteinases. Therefore, epiretinal fibrovascular membranes from 16 patients with PDR were studied by immunohistochemical analysis. Immunoreactivity for the studied ADAMTS proteinases was observed in all membranes. Figure 4 shows representative immunohistochemical analysis results for ADAMTS-4 expression. Immunoreactivity for ADAMTS-4 was noted in endothelial cells lining new blood vessels (Figure 4A,B) and stromal cells. Stromal cells were spindle-shaped myofibroblasts expressing α-SMA according to their morphology (Figure 4C), leukocytes co-expressing the leukocyte common antigen CD45 (Figure 5A), monocytes/macrophages and neutrophil granulocytes co-expressing CD68 (Figure 5B).

### 2.5. Expression of MMP-15 in Epiretinal Fibrovascular Membranes from Patients with PDR

**Figure 6:**
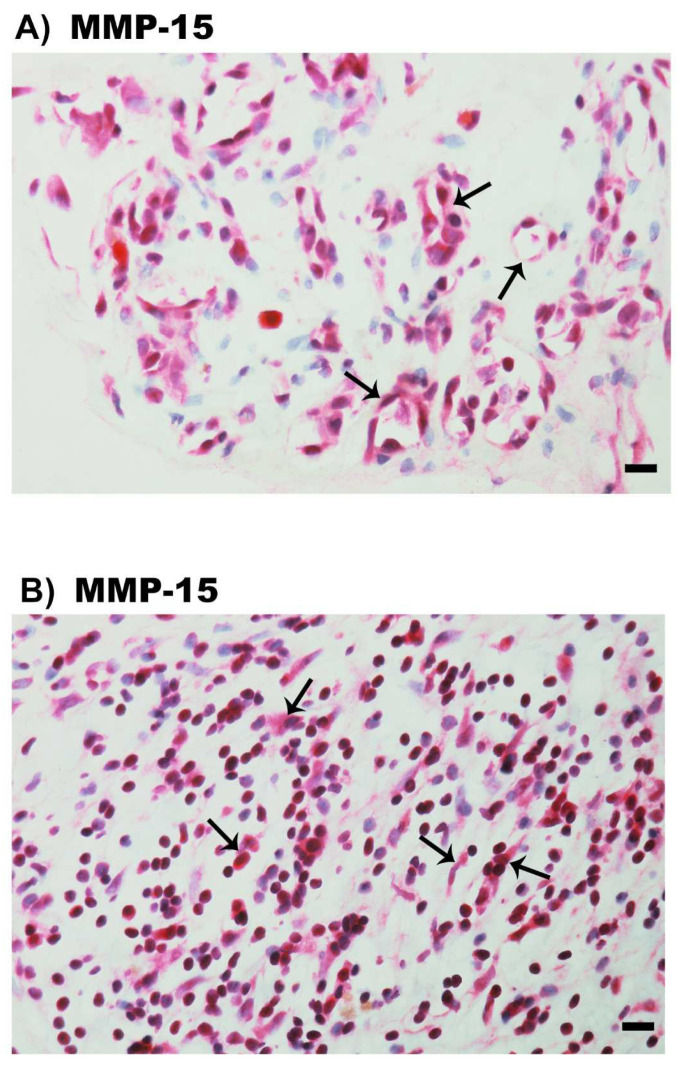

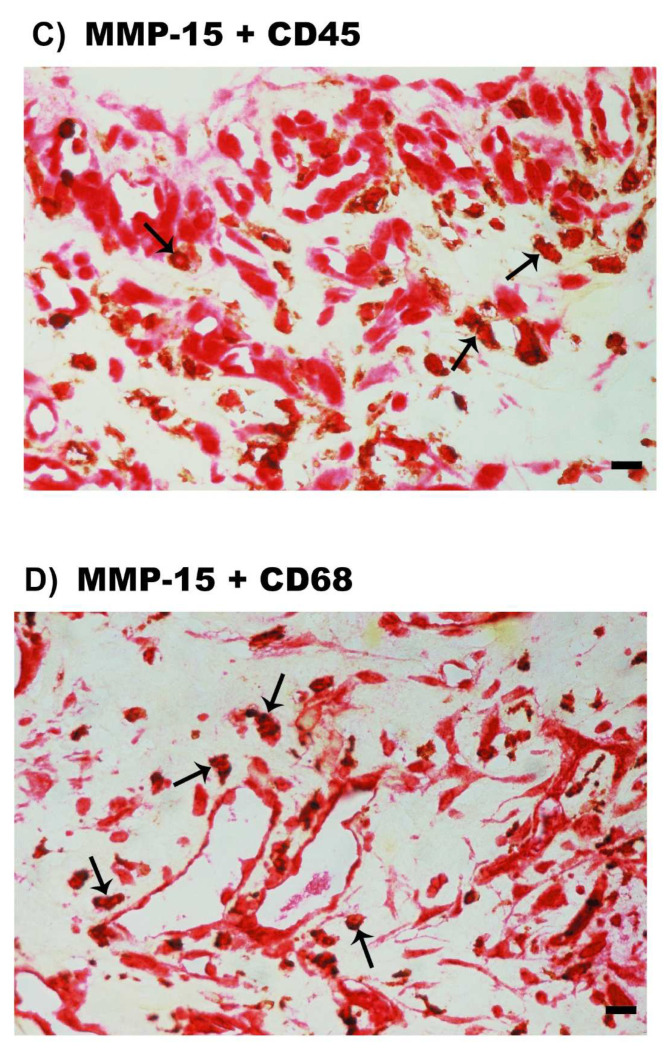
Immunohistochemical staining of epiretinal fibrovascular membranes from patients with proliferative diabetic retinopathy. Immunohistochemical staining for matrix metalloproteinase (MMP) -15 showing immunoreactivity in vascular endothelial cells (arrows) (**A**). Immunoreactivity for MMP-15 was also detected in stromal spindle-shaped myofibroblasts (arrows) (**B**). Double immunohistochemical staining for MMP-15 (red) and CD45 (brown) (**C**) or CD68 (brown) (**D**) demonstrating co-expression in stromal cells (arrows). No counterstain to visualize the cell nuclei was applied (arrows) (scale bar, 10 µm).

Since the activation of ADAMTS proteinases requires processing by MMP-15 [23], we evaluated the expression of MMP-15 in epiretinal fibrovascular membranes from patients with PDR. MMP-15 immunoreactivity was observed in vascular endothelial cells (Figure 6A) and stromal cells (Figure 6B). Stromal cells were spindle-shaped cells (Figure 6B) expressing α-SMA (Figure 3D), leukocytes co-expressing CD45 (Figure 6C), monocytes/macrophages and neutrophil granulocytes co-expressing CD68 (Figure 6D).

### 2.6. Expression of α-SMA, the Leukocyte Common Antigen CD45 and the Monocyte/Macrophage Marker CD68 in Epiretinal Fibrocellular Membranes from Patients with PVR

**Figure 7:**
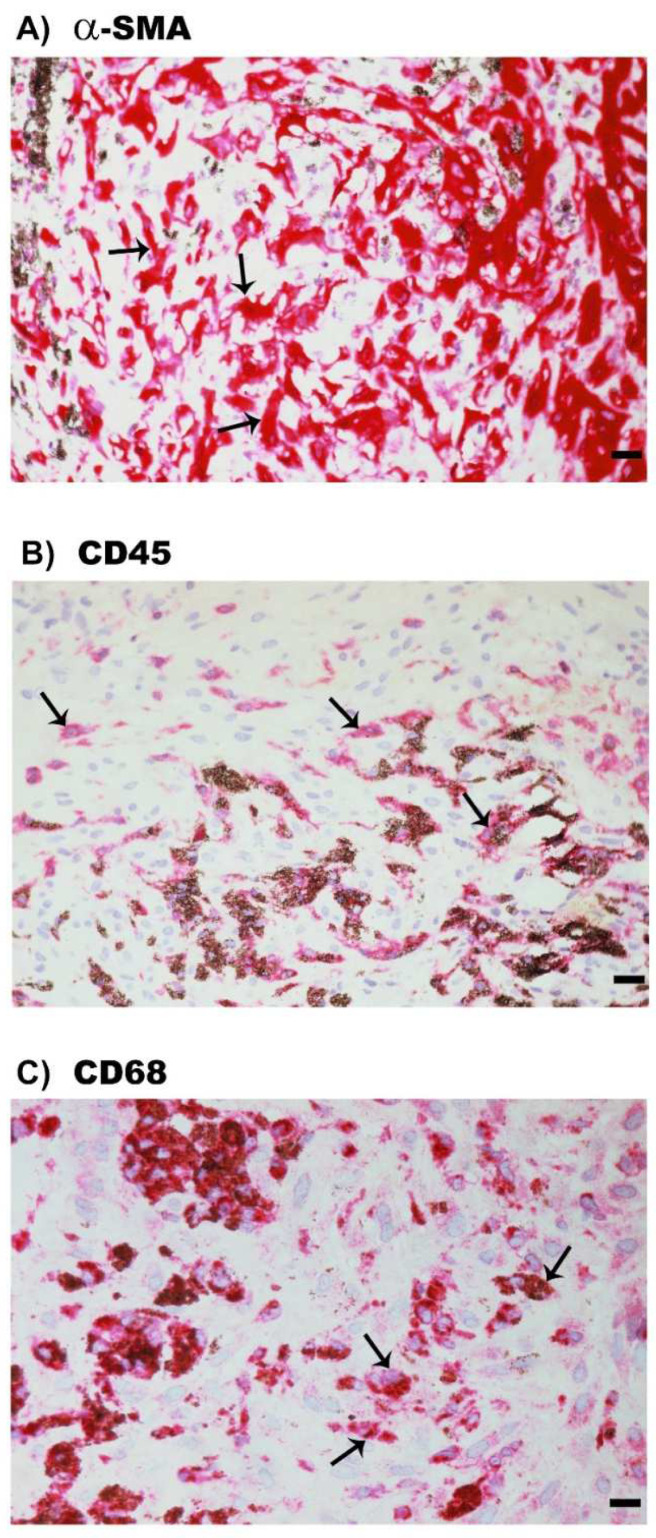
Immunohistochemical staining of epiretinal fibrocellular membranes from patients with proliferative vitreoretinopathy. Immunohistochemical staining for α-smooth muscle actin (α-SMA) showing immunoreactivity in spindle-shaped myofibroblasts (arrows) (**A**). Immunohistochemical staining for CD45 showing immunoreactivity in leukocytes (arrows) (**B**). Immunohistochemical staining for CD68 showing immunoreactivity in monocytes/macrophages (arrows) (**C**) (scale bar, 10 µm).

For comparison, we examined epiretinal fibrocellular membranes from patients with retinal detachment complicated by PVR. All membranes showed spindle-shaped myofibroblasts expressing α-SMA (Figure 7A). Leukocytes expressing CD45 (Figure 7B), monocytes/macrophages expressing and neutrophil granulocytes CD68 (Figure 7C) were detected in all membranes.

### 2.7. Expression of ADAMTS Proteinases in Epiretinal Fibrocellular Membranes from Patients with PVR

**Figure 8:**
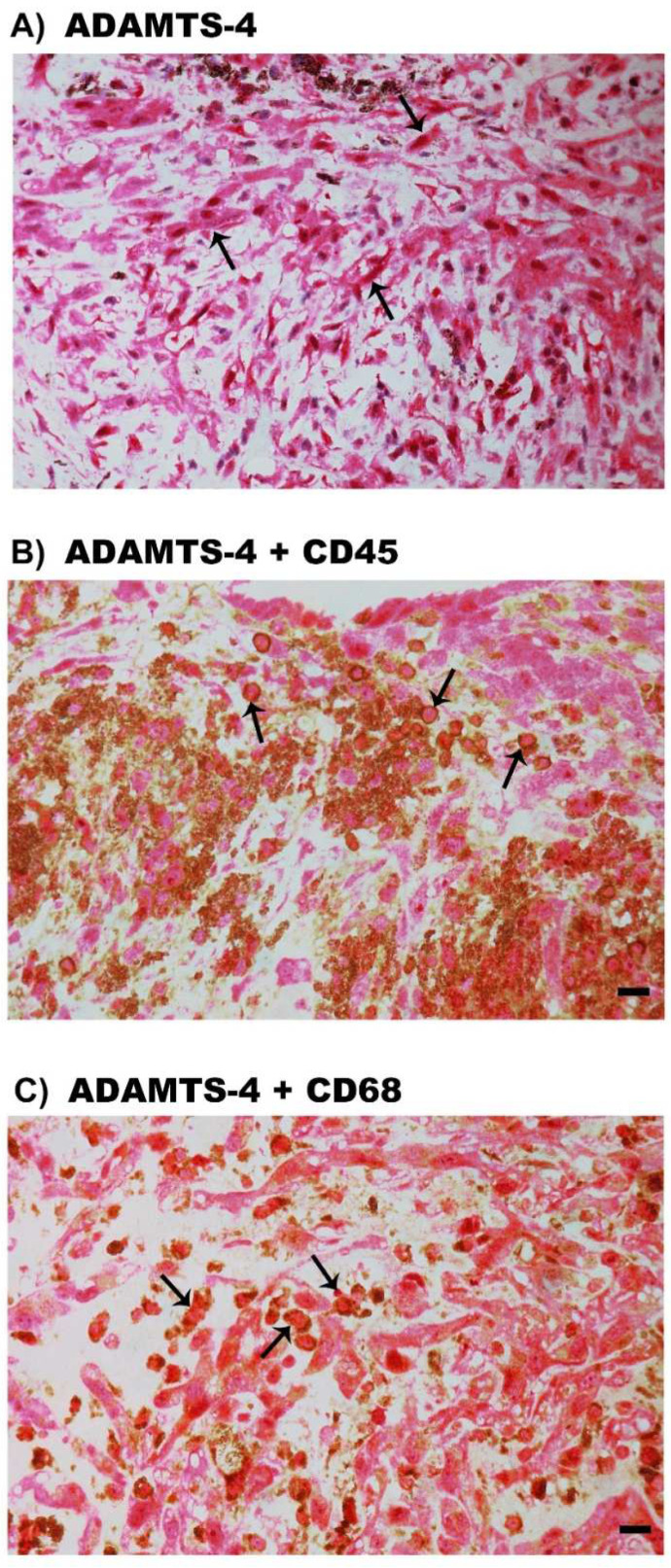
Immunohistochemical staining of epiretinal fibrocellular membranes from patients with proliferative vitreoretinopathy. Immunohistochemical staining for ADAMTS-4 showing immunoreactivity in spindle-shaped myofibroblasts (arrows) (**A**). Double immunohistochemistry for ADAMTS-4 (red) and CD45 (brown) (**B**) or CD68 (brown) (**C**) showing co-expression. No counterstain to visualize the cell nuclei was applied (arrows) (scale bar, 10 µm).

Immunohistochemical staining for ADAMTS-1, ADAMTS-13 and MMP-15 revealed no immunoreactivity. Immunostaining for ADAMTS-2, ADAMTS-4 and ADAMTS-5 was detected in spindle-shaped α-SMA-expressing myofibroblasts. Figure 8A shows representative immunohistochemical analysis results for ADAMTS-4 expression in myofibroblasts. In addition, double-labeling experiments showed that cells expressing ADAMTS-4 co-expressed CD45 (Figure 8B) and CD68 (Figure 8C).

### 2.8. Correlations between ADAMTS Proteinases Expression and Angiogenic Activity in Epiretinal Fibrovascular Membranes from Patients with PDR

Table 1 shows the mean numbers of immunoreactive blood vessels and stromal cells expressing the studied ADAMTS proteinases. The mean numbers of cells expressing ADAMTS proteinases differed significantly (*p* < 0.001; ANOVA). Pairwise comparisons (independent t-test) indicated that the mean numbers of cells expressing ADAMTS-4 were significantly higher than the mean numbers of cells expressing ADAMTS-1 (*p* < 0.001), ADAMTS-2 (*p* = 0.015), ADAMTS-5 (*p* = 0.01) and ADAMTS-13 (*p* < 0.001). The mean number of cells expressing ADAMTS-13 was significantly lower than the mean number of cells expressing ADAMTS-1 (*p* = 0.013), ADAMTS-2 (*p* = 0.014) and ADAMTS-5 (*p* = 0.038). Similarly, the mean number of blood vessels that were immunoreactive for ADAMTS-4 were significantly higher than the mean number of blood vessels that were immunoreactive for ADAMTS-1 (*p* = 0.002), ADAMTS-5 (*p* = 0.009) and ADAMTS-13 (*p* < 0.001). The mean number of blood vessels that were immunoreactive for ADAMTS-13 were significantly lower than the mean number of blood vessels expressing ADAMTS-1, ADAMTS-2 and ADAMTS-5 (*p* < 0.001 for all comparisons). The number of blood vessels that were immunoreactive for CD31, reflecting the angiogenic activity of PDR epiretinal fibrovascular membranes, correlated significantly (Pearson correlation coefficients) with the numbers of stromal cells expressing ADAMTS-1 (r = 0.597; *p* = 0.015) and ADAMTS-4 (r = 0.530; *p* = 0.035).

### 2.9. Effect of Diabetes on the Retinal Expression of ADAMTS Proteinases and MMP-15 in Experimental Rats

**Figure 9:**
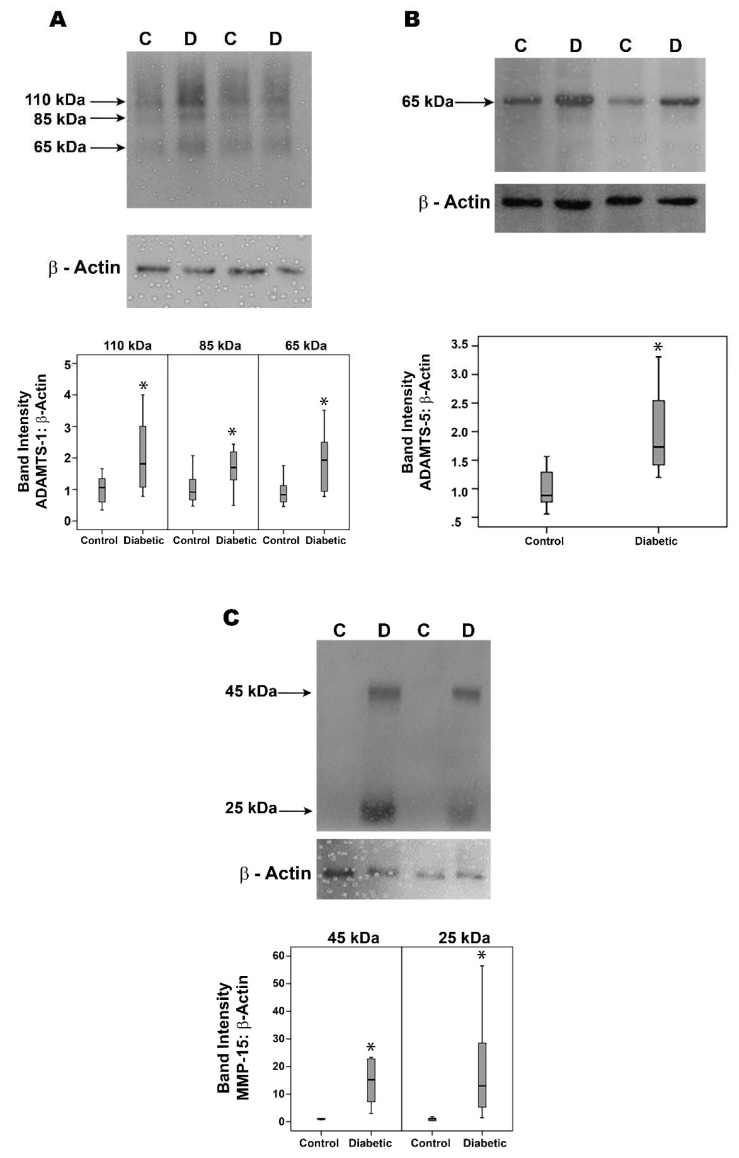
ADAMTS-1 (**panel A**), ADAMTS-5 (**panel B**) and matrix metalloproteinase (MMP-15) (**panel C**) expression levels in the retinas of diabetic rats. Protein expression was determined by Western blot analysis on lysates of retinas from 4-week diabetic rats (D) (*n* = 12) and nondiabetic control retinas (C) (*n* = 12). After determination of the intensity of the protein bands, intensities were adjusted to those of β-actin in the sample. Results are expressed as a median (interquartile range) (* *p* < 0.05; Mann–Whitney test).

Western blot analysis demonstrated that ADAMTS-1 was expressed as three protein bands at approximately 110 kDa, 85 kDa and 65 kDa (Figure 9A). The predominant ADAMTS-1 proteoform was found at 110 kDa, indicating that ADAMTS-1 in the retina was largely the proform. ADAMTS-2 immunoreactivities were expressed as three protein bands at approximately 58 kDa, 45 kDa and 35 kDa. The predominant ADAMTS-2 proteoform was found at 58 kDa (data not shown). ADAMTS-4 immunoreactivities were expressed as two protein bands at 70 kDa and 45 kDa. The predominant ADAMTS-4 proteoform was found at 45 kDa indicating that ADAMTS-4 in the retina was largely the processed form (data not shown). ADAMTS-5 immunoreactivity was expressed as one protein band at approximately 65 kDa (Figure 9B). ADAMTS-13 immunoreactivities were expressed as three protein bands. The approximate molecular weights of these bands were around 60 kDa, 35 kDa and 25 kDa. Most of the ADAMTS-13 immunoreactivity appeared at the level of 35 kDa form (data not shown). MMP-15 immunoreactivities were expressed as two protein bands at approximately 45 kDa and 25 kDa (Figure 9C). Densitometric analysis of the bands demonstrated increased ADAMTS-1 (Figure 9A), ADAMTS-5 (Figure 9B) and MMP-15 (Figure 9C) protein levels in the retina of rats after 4 weeks of streptozotocin-induced diabetes, whereas the expression of ADAMTS-2, ADAMTS-4 and ADAMTS-13 did not differ significantly between nondiabetic controls and diabetic rat retinas (data not shown).

### 2.10. Human Retinal Müller Glial Cells and Human Retinal Microvascular Endothelial Cells Constitutively Express ADAMTS-1, -2, -4, -5 and -13 but Not MMP-15

**Figure 10:**
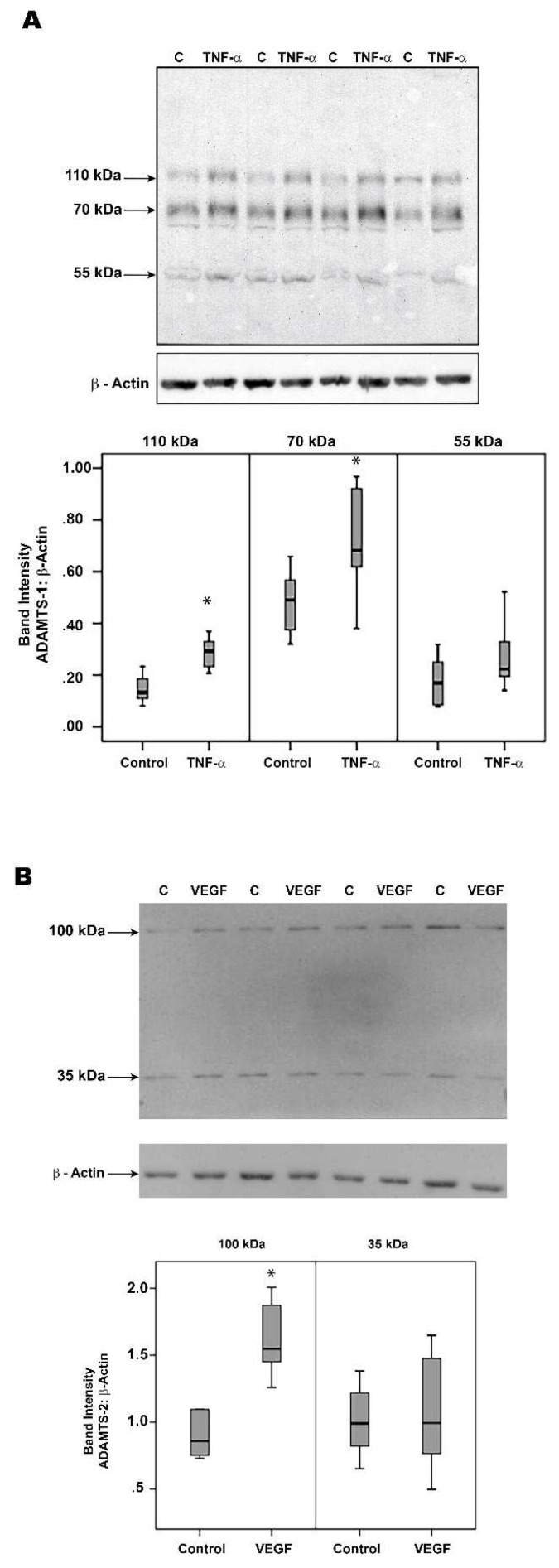
Müller cells were left untreated (C) or treated with tumor necrosis factor-α (TNF-α) (50 ng/mL) or vascular endothelial growth factor (VEGF) (50 ng/mL) for 24 h. ADAMTS-1 (**panel A**) and ADAMTS-2 (**panel B**) expression levels in the cell lysates were determined by Western blot analysis. Results are expressed as a median (interquartile range) from three different experiments (* *p* < 0.05; Mann–Whitney test).

Western blot analysis of cell lysates demonstrated that retinal Müller glial cells express ADAMTS-1, -2, -4, -5 and -13 but not MMP-15. ADAMTS-1 immunoreactivities were expressed as three protein bands at approximately 110 kDa, 70 kDa and 55 kDa. The predominant ADAMTS-1 proteoform was found at 70 kDa (Figure 10A). ADAMTS-2 immunoreactivities were expressed as two protein bands at approximately 100 kDa and 35 kDa. As shown in Figure 10B, the predominant proteoform was found at 100 kDa. ADAMTS-4 immunoreactivities were expressed as three protein bands at 95 kDa, 70 kDa and 55 kDa. The predominant ADAMTS-4 was around 70 kDa (data not shown). ADAMTS-5 immunoreactivities were expressed as three protein bands at approximately 65 kDa, 40 kDa and 30 kDa. Most of the ADAMTS-5 immunoreactivity appeared at the level of 65 kDa form (data not shown). ADAMTS-13 immunoreactivities were expressed as two protein bands at approximately 60 kDa and 45 kDa (data not shown). The treatment of Müller cells with the proinflammatory cytokine TNF-α induced the significant upregulation of the ADAMTS-1 proteoforms 110 kDa and 70 kDa, compared to the untreated control (Figure 10A). However, TNF-α did not affect the expression of ADAMTS-2, -4, -5 and -13, compared to the untreated control (data not shown). The treatment of Müller cells with VEGF induced the significant upregulation of the ADAMTS-2 proteoform 100 kDa, compared to the untreated control (Figure 10B). However, VEGF did not affect the expression of the other ADAMTS proteinases (data not shown).

With the use of Western blot analysis, we confirmed that HRMECs express ADAMTS-1, -2, -4, -5 and -13. However, HRMECs did not express MMP-15. ADAMTS-1 was expressed as three protein bands at approximately 110 kDa, 70 kDa and 55 kDa. The predominant ADAMTS-1 proteoform was found at 110 kDa. ADAMTS-2 immunoreactivity was expressed as one protein band at approximately 35 kDa. ADAMTS-4 immunoreactivities were expressed as three protein bands at 95 kDa, 70 kDa and 55 kDa. The predominant ADAMTS-4 proteoform was found at 55 kDa. ADAMTS-5 immunoreactivities were expressed as two protein bands at 65 kDa and 37 kDa. The predominant ADAMTS-5 proteoform was found at 37 kDa. ADAMTS-13 immunoreactivities were expressed as two protein bands at 60 and 45 kDa. Most of the ADAMTS-13 immunoreactivity appeared at the level of 60 kDa. Densitometric analysis of the bands demonstrated that treatment with TNF-α or VEGF did not affect the expression of the studied ADAMTS proteinases as compared to untreated control (data not shown).

### 2.11. The NF-κB Inhibitor BAY11-7085 Attenuates the TNF-α-and-VEGF-Induced Upregulation of ADAMTS-1 and -4 in Human Retinal Microvascular Endothelial Cells and Human Retinal Müller Glial Cells

**Figure 11:**
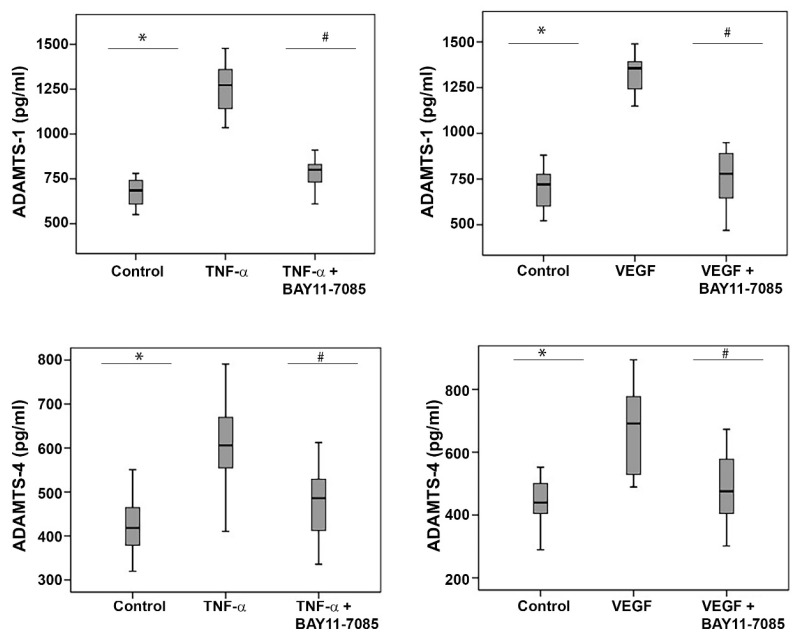
Human retinal microvascular endothelial cells (HRMECs) were left untreated or treated with tumor necrosis factor-α (TNF-α) (50 ng/mL), vascular endothelial growth factor (VEGF) (50 ng/mL) and TNF-α (50 ng/mL) plus BAY11-7085 (10 µM) or VEGF (50 ng/mL) plus BAY11-7085 (10 µM) for 24 h. The levels of secreted ADAMTS-1 and ADAMTS-4 were quantified in the culture media by ELISA. Results are expressed as a median (interquartile range) from three different experiments. Kruskal–Wallis and Mann–Whitney tests were used for comparisons between three groups and two groups, respectively. * *p* < 0.05 compared with values obtained from untreated cells. # *p* < 0.05 compared with TNF-α plus BAY11-7085 or VEGF plus BAY11-7085-treated cells.

**Figure 12:**
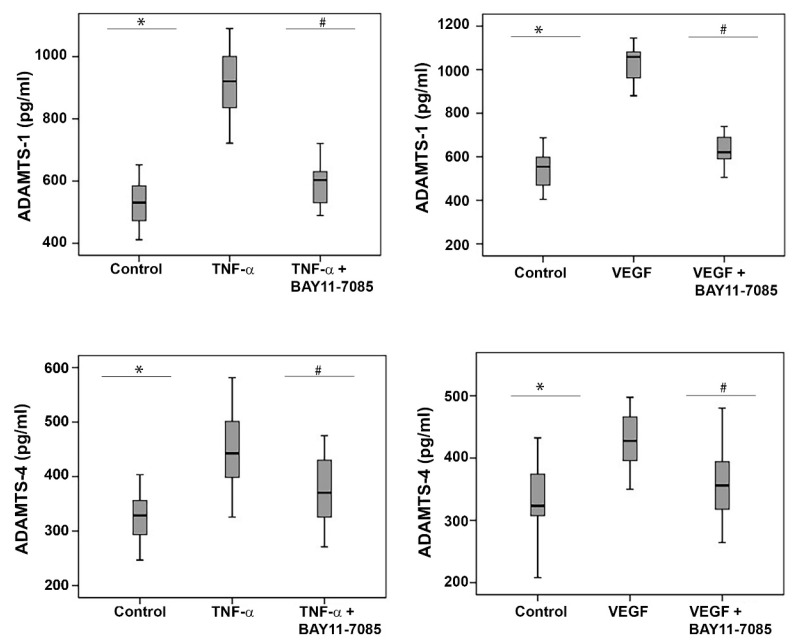
Müller cells were left untreated or treated with tumor necrosis factor-α (TNF-α) (50 ng/mL), vascular endothelial growth factor (VEGF) (50 ng/mL) and TNF-α (50 ng/mL) plus BAY11-7085 (10 µM) or VEGF (50 ng/mL) plus BAY11-7085 (10 µM) for 24 h. The levels of secreted ADAMTS-1 and ADAMTS-4 were quantified in the culture media by ELISA. Results are expressed as a median (interquartile range) from three different experiments. Kruskal–Wallis and Mann–Whitney tests were used for comparisons between three groups and two groups, respectively. * *p* < 0.05 compared with valves obtained from untreated cells. # *p* < 0.05 compared with TNF-α plus BAY11-7085 or VEGF plus BAY11-7085-treated cells.

To investigate the involvement of the inflammatory transcription factor NF-κB in the TNF-α-and-VEGF-induced upregulation of ADAMTS-1 and -4, we treated HRMECs and Müller glial cells with TNF-α (50 ng/mL), VEGF (50 ng/mL) and TNF-α (50 ng/mL) plus BAY11-7085 (10 µM) or VEGF (50 ng/mL) plus BAY11-7085 (10 µM). ELISA analysis revealed that the treatment of HRMECs (Figure 11) and Müller cells (Figure 12) with the proinflammatory cytokine TNF-α and the proangiogenic factor VEGF significantly increased the levels of secreted ADAMTS-1 and -4 in the culture medium, compared to the untreated control. Co-treatment with TNF-α plus BAY11-7085 or VEGF plus BAY11-7085 significantly attenuated the TNF-α-and-VEGF-induced upregulation of ADAMTS-1 and -4 in HRMECs (Figure 11) and Müller cells (Figure 12).

## 3. Discussion

In the current study, we demonstrated that the expression levels of ADAMTS-1, -2, -4, -5 and -13 were upregulated in vitreous fluid samples and were expressed in epiretinal fibrovascular membranes from patients with PDR. With the use of immunohistochemical analysis, we demonstrated that the studied ADAMTS proteinases were specifically localized in the endothelial cells of pathologic new blood vessels, monocytes/macrophages and myofibroblasts in epiretinal fibrovascular membranes from patients with PDR. We also demonstrated that, of the 5 ADAMTS proteinases analyzed, ADAMTS-4 was expressed in the highest number of stromal cells in epiretinal membranes. In addition, our analysis showed significant positive correlations between the degree of angiogenic activity in PDR epiretinal fibrovascular membranes and the levels of ADAMTS-1 and ADAMTS-4 cellular expression. Our findings suggest a predominant role of ADAMTS-4 and ADAMTS-1 as proteoglycan-degrading enzymes over other studied ADAMTS members in pathologic angiogenesis in PDR. Furthermore, among the examined ADAMTS proteinases, ADAMTS-1 and ADAMTS-5 were upregulated in the retinas of diabetic rats. Similarly, a previous study demonstrated increased ADAMTS-1 expression, along with VEGF expression, in the retina of a mouse model of oxygen-induced retinal ischemia and neovascularization [24].

ADAMTS proteinases are synthesized as inactive zymogens, which require activation via the proteolytic removal of the prodomain, a key posttranslational processing step, before they can exert catalytic activity. Secreted pro-ADAMTS proteinases are intracellularly processed by the proprotein convertase furin in the trans-Golgi network and by MMP-15 in the pericellular space to generate mature proteolytically active forms [16,18,19,20,23]. Furin also exists as a soluble/secreted or shed form that could play a role in processing extracellular precursor proteins [16,23]. We demonstrated that levels of furin [25] and MMP-15 were upregulated in vitreous fluid samples from patients with PDR and in the retina of diabetic rats. Using immunohistochemistry, we demonstrated that the studied ADAMTS proteinase and their activating enzymes furin [25] and MMP-15 were co-expressed in the monocytes/macrophages, myofibroblasts and endothelial cells of pathologic new blood vessels in epiretinal fibrovascular membranes from patients with PDR. The co-expression of ADAMTS proteinases and their activating enzymes MMP-15 and furin in the ocular microenvironment of patients with PDR suggests the conversion of the ADAMTS proteinase proforms into processed forms and is a proxy for their enzymatic activity. The detection of processed forms of ADAMTS proteinases by Western blot analysis in the vitreous fluid from patients with PDR at higher levels than in control vitreous samples suggested efficient proteolytic removal of the prodomain prior to or after secretion into the vitreous fluid and some association with the disease processes. Whether increased catalytic activity in the conversion of specific substrates, such as versican and biglycan, is cause or consequence in PDR and whether these ADAMTS proteinases have detrimental or restorative effects on the disease remain to be determined, e.g., by animal model studies. The approach of using Western blot analysis has limitations for interpretation of data obtained with clinical data. However, it is clear that the commonly used ELISA technique also has the limitation that single quantitative numbers are given for single samples, whereas such samples ofren contain mixtures of various proteoforms, including inactive pro-enzymens, activated enzymes and inactive degradation products. Further standardizations for the purpose of diagnosis, prognosis and follow-up of therapy on the basis of specific proteoforms as biomarkers are therefore recommended.

In an in vivo model of VEGF-induced angiogenesis, Fu et al. [13] demonstrated the increased expression of ADAMTS-1 with one of its activators, MMP-15, associated with early striking degradation of the vascular basement membrane versican. Furthermore, VEGF induced the expression of ADAMTS-1 and MMP-15 in cultured endothelial cells. Such observations suggest that versican processing by ADAMTS-1 is involved in the pathogenesis of pathologic angiogenesis. During endothelial cell tubulogenesis in vitro, ADAMTS-4 was relatively abundant, while the expression levels of ADAMTS-1 and -5 were low. These findings suggest a predominant role for ADAMTS-4 over other ADAMTS members during angiogenesis. In addition, tubulogenic cultures contained biglycan cleavage products [14]. These findings suggest that ADAMTS-4 and its proteoglycan substrate biglycan are involved in tubulogenesis by endothelial cells.

In this study, we showed that the expression of the proteoglycanases ADAMTS-1, -4 and -5 in the vitreous fluid samples from patients with PDR associated with the proteolytic fragments of the proteoglycan substrates versican and biglycan. These findings suggest an enzyme/substrate relationship between these molecules in the pathogenesis of PDR initiation and progression and that the degradation of versican, which is present in the vasculature, may participate in the breakdown of the blood–retinal barrier. It has been suggested that versican plays an important role in maintaining the structural and functional integrity of the vascular wall, and its degradation may be responsible for vascular diseases [26]. Consistent with the results of our study, previous reports showed the degradation of versican in aortic tissues from patients with thoracic aortic aneurysms and dissections [15,27].

ADAMTS proteinases were reported to have both pro- and anti-angiogenic activities. A possible mechanism for the dual role of ADAMTS-1 in angiogenesis progression is the differential activity of full-length versus N- or C-terminally cleaved forms of the protein. Thus, full-length ADAMTS-1 has been demonstrated to promote the progression of tumor angiogenesis, but the truncated forms were able to suppress angiogenesis and tumor growth [28,29]. In addition, Krambert et al. [30] demonstrated a dual function of ADAMTS-1 in the context of fibroblast and endothelial cell migration. Low concentrations of ADAMTS-1 stimulate fibroblast and endothelial cell migration via its proteolytic activity, whereas high concentrations inhibit this process. ADAMTS-4 has also been demonstrated to have dual functions in cancer. The full length ADAMTS-4 and its catalytically active N-terminal 53 kDa (auto)catalytic fragment both promote tumor growth and angiogenesis. In contrast, the overexpression of its catalytically inactive mutant or truncated fragments containing only the C-terminal ancillary domains suppresses tumor growth and angiogenesis [17]. Interestingly, both full-length and cleaved fragments of ADAMTS-1 and ADAMTS-4 were identified in vitreous fluid from patients with PDR. Similarly, a dual role of ADAMTS-13 in angiogenesis was reported. The treatment of endothelial cells with exogenous recombinant full-length ADAMTS-13 promoted endothelial cell tube formation, proliferation and migration. In contrast, ADAMTS-13 inhibited VEGF-induced angiogenesis [31]. These findings suggest that the role of ADAMTS proteinases in angiogenesis might depend on the local environmental setting.

Leukocytes in fibrovascular epiretinal membranes from patients with PDR play critical roles in the initiation and progression of PDR by producing proinflammatory factors, proangiogenic factors and proteolytic enzymes [1,2,3,4,5,6]. In the present study, we demonstrated for the first time that the studied ADAMTS proteinases were co-localized with monocytes/macrophages in PDR epiretinal fibrovascular membranes. These findings suggest that inflammatory cells are a major source of these proteinases in the ocular microenvironment of patients with PDR. In several studies, it was demonstrated that the dysregulation of ADAMTS expression may contribute to tissue destruction, inflammation and the development of vascular diseases, such as atherosclerosis [32,33] and thoracic aortic aneurysms and dissections [15,27]. ADAMTS-1, -4 and -5 were found to be highly expressed in macrophages in human atherosclerotic plaques [32,33] and in aortic tissues from patients with thoracic aortic aneurysms and dissections [15,27]. In addition, ADAMTS-1 expression was upregulated in wounded skin and macrophages were the source of ADAMTS-1 in early wounds, whereas keratinocytes and fibroblasts produced this proteinase at later stages of wound healing [30].

ADAMTS proteinases may influence the functioning of monocytes/macrophages in several ways. For instance, ADAMTS-1, -4 and -5 are expressed in monocytes and their levels increased significantly during differentiation into macrophages [32,33,34]. Several reports suggest a role for ADAMTS-4 in macrophage invasion in vitro [15,27]. In addition, it was demonstrated that the ADAMTS proteinases are inflammation-associated proteins. ADAMTS-1 was potently induced in endothelial cells by lipopolysaccharide or the proinflammatory cytokine TNF-α [24,35]. The proinflammatory cytokine interleukin-1β (IL-1β) induced ADAMTS-4 and -5 in synovial fibroblasts [36]. The proinflammatory cytokines interferon-γ, TNF-α and IL-1β induced the expression of ADAMTS-4 in macrophages [32].

HRMECs and retinal Müller glial cells are major cell types that actively participate in diabetes-induced inflammatory and angiogenic reactions in the retina [1,2,3,4,5,6,25,37]. VEGF is considered the most potent proangiogenic factor in PDR [38,39]. Müller cells, a major source of VEGF secretion, contribute to the development of pathological retinal neovascularization [37]. In the present study, we reported the capability of the proinflammatory cytokine TNF-α and the proangiogenic factor VEGF to target HRMECs and Müller cells and to induce the synthesis and secretion of ADAMTS-1 and ADAMTS-4. This is in line with previous studies documenting that endothelial cells produce ADAMTS-1, -4, -5 [13,40] and -13 [41] and the capacity of VEGF [13,24] and TNF-α [24,25] to upregulate the expression of ADAMTS-1 in cultured endothelial cells. We also demonstrated that the inhibition of the inflammatory transcription factor NF-κB significantly attenuated TNF-α-and-VEGF-induced upregulation of ADAMTS-1 and ADAMTS-4 in the culture medium of HRMECs and Müller cells. This is in line with previous studies documenting that the proinflammatory cytokines TNF-α and IL-1β can promote ADAMTS-4 expression through the activation of the NF–κB signaling pathway [42,43].

## 4. Materials and Methods

### 4.1. Patient Samples

Undiluted vitreous fluid samples (0.3–0.6 mL) were obtained from 16 patients with PDR during pars plana vitrectomy, for the treatment of tractional retinal detachment, and/or nonclearing vitreous hemorrhage. We compared the samples from diabetic patients with those of a clinical control cohort. The control group consisted of 16 patients who had undergone vitrectomy for the treatment of rhegmatogenous retinal detachment with no proliferative vitreoretinopathy (PVR). Control subjects were clinically checked to be free from diabetes or other systemic disease. Vitreous samples were collected undiluted by manual suction into a syringe through the aspiration line of vitrectomy, before opening the infusion line. The samples were centrifuged (700× *g* for 10 min, 4 °C), and the supernatants were aliquoted and frozen at −80 °C until assay. Epiretinal fibrovascular membranes were obtained from 16 patients with PDR during pars plana vitrectomy for the repair of tractional retinal detachment. For comparison, epiretinal fibrocellular membranes were obtained from 10 patients without diabetes undergoing vitreoretinal surgery for the treatment of retinal detachment complicated by PVR. Membranes were fixed for 2 h in 10% formalin solution and embedded in paraffin.

### 4.2. Immunohistochemical Staining of Human Epiretinal Membranes and Quantifications

For CD31, α-SMA and MMP-15 detection, antigen retrieval was performed by boiling the sections in a citrate-based buffer (pH 5.9–6.1) (BOND Epitope Retrieval Solution 1; Leica Biosystems, IL, USA) for 10 min. For CD45, CD68, ADAMTS-1, ADAMTS-2, ADAMTS-4, ADAMTS-5 and ADAMTS-13 detection, antigen retrieval was performed by boiling the sections in Tris/EDTA buffer (pH 9) (BOND Epitope Retrieval Solution 2; Leica) for 20 min. Subsequently, the sections were incubated for 60 min with mouse monoclonal anti-CD31 (ready-to-use; clone JC70A; Dako, Glostrup, Denmark), mouse monoclonal anti-CD45 (ready-to-use; clones 2B11+PD7/26; Dako), mouse monoclonal antibody against α-SMA (ready-to-use: clone 1A4; Dako), mouse monoclonal anti-CD68 antibody (ready-to-use; clone KP1; Dako), rabbit polyclonal antibody against ADAMTS-1 (1:100; ab39194, Abcam, Cambridge, UK), rabbit polyclonal antibody against ADAMTS-2 (1:400; ab125226, Abcam), rabbit polyclonal antibody against ADAMTS-4 (1:200; ab185722, Abcam), rabbit polyclonal antibody against ADAMTS-5 (1:100; ab41037, Abcam), rabbit polyclonal antibody against ADAMTS-13 (1:400; ab71550, Abcam) and rabbit polyclonal antibody against matrix metalloproteinase (MMP)-15 (1:100; ab135562, Abcam). Optimal working conditions for the antibodies were determined in pilot experiments on human heart, kidney, liver and spleen sections. The sections were then incubated for 20 min with an IgG against the primary antibody and conjugated with alkaline phosphatase. The reaction product was visualized by incubation for 15 min with the Fast Red chromogen, resulting in bright red immunoreactive sites. The slides were then faintly counterstained with Mayer’s hematoxylin (BOND Polymer Refine Red Detection Kit; Leica).

To identify the phenotype of cells expressing ADAMTS proteinases and MMP-15, sequential double immunohistochemistry was performed. The sections were incubated with the first primary antibodies (anti-CD45 or anti-CD68) and subsequently treated with a peroxidase-conjugated secondary antibody to define the leukocytes. The resulting immune complexes were visualized by an enzymatic reaction of the 3, 3′-diaminobenzidine tetrahydrochloride substrate, yielding brown precipitates. Incubation with anti-ADAMTS proteinases and MMP-15 as primary antibodies was followed by treatment with an alkaline phosphatase-conjugated secondary antibody and Fast Red reactions, as indicated above. No counterstain was applied. In negative control experiments for the estimation of background staining, the incubation step with primary antibodies was omitted from the staining protocol. Instead, the ready-to-use DAKO Real antibody Diluent (Agilent Technologies Product Code 52022) was applied.

The level of vascularization in epiretinal membranes was determined by the immunodetection of the vascular endothelium marker CD31. Immunoreactive blood vessels and cells were counted in five representative fields, with the use of an eyepiece with a calibrated grid in combination with the 40× objective. These representative fields were selected based on the presence of immunoreactive blood vessels and cells. With the used magnification and calibration, immunoreactive blood vessels and cells present in an area of 0.33 mm × 0.22 mm were counted.

### 4.3. Induction of Streptozotocin-Induced Diabetes in Rats

Adult male Sprague Dawley rats of 8–9 weeks of age (200–220 g) were fasted overnight, and a single-bolus dose of streptozotocin of 65 mg/ kg in 10 mM sodium citrate buffer, pH 4.5, (Sigma, St. Louis, MO, USA) was injected intraperitoneally. Equal volumes of citrate buffer were injected in age-matched control rats. Rats were considered diabetic if their blood glucose levels were in excess of 250 mg/dL. After 4 weeks of diabetes, the rats were euthanized by an overdose of chloral hydrate, the eyes were removed, and retinas were isolated and frozen immediately in liquid nitrogen and stored at −80 °C until analyzed. Similarly, retinas were obtained from age-matched nondiabetic control rats. We used this standard experimental rat model to show the effect of hyperglycemia on the retina and to illustrate similar changes in the parameters found in humans with diabetes. However, we recognize that, in the rat, we are limited to only studying short-term (mainly inflammatory) effects, whereas in the human eyes, the observations may be developing over several years of disease (long-term). In the used animal model, streptozotocin (STZ) destroys pancreatic island β cells and induces experimental diabetes. Adult rats treated with a single dose of STZ demonstrating hyperglycemia within 48 h are widely used as a model of insulin-dependent diabetes mellitus. Streptozotocin-induced diabetic rats demonstrate characteristics of the nonproliferative diabetic retinopathy that occurs in humans, such as inflammation and increased vascular permeability resulting from the breakdown of the blood–retinal barrier (BRB). The breakdown of the BRB was observed in this animal model as early as 2 weeks following diabetes induction [44,45,46,47,48].

### 4.4. Human Retinal Müller Glial Cell and Human Retinal Microvascular Endothelial Cell Cultures

Human retinal Müller glial cells (MIO-M1) (a generous gift from Prof. A. Limb, Institute of Ophthalmology, University College London, UK) were cultured in Dulbecco’s Minimal Essential Medium (DMEM) containing 1 g/L glucose with 10% (*v*/*v*) fetal bovine serum and 1% penicillin/streptomycin. Confluent cells were starved overnight in serum-free DMEM to minimize the effects of serum. Subsequently, the cell cultures were either left untreated or stimulated for 24 h.

Human retinal microvascular endothelial cells (HRMECs) were purchased from Cell Systems Corporation (Kirkland, WA, USA) and maintained in complete serum-free media (Cat No SF-4Z0–500, Cell System Corporation) supplemented with “Rocket Fuel” (Cat No SF-4Z0-500, Cell System Corporation), “Culture Boost” (Cat No 4CB-500, Cell System Corporation) and antibiotics (Cat No 4Z0–643, Cell System Corporation) at 37 °C in a humidified atmosphere with 5% CO2. We used HRMECs up to passage 8 for all of our experiments. Cell cultures at about 80% confluency were starved in a minimal medium (medium supplemented with 0.25% “Rocket Fuel” and antibiotics) overnight to eliminate any residual effects of growth factors.

The following stimuli were used: 50 ng/mL recombinant human tumor necrosis factor-alpha (TNF-α) (Cat No 210-TA, R&D Systems, Minneapolis, MN, USA) or 50 ng/mL recombinant human vascular endothelial growth factor (VEGF) (Cat No 293-VE-050, R&D Systems) in the absence or presence of the nuclear factor-kappa B (NF-κB) inhibitor BAY11-7085 (10 µM) (Cat No sc-202490, Santa Cruz Biotechnology Inc., Santa Cruz, CA, USA).

After 24 h, cell supernatants were collected and processed for ELISA analysis. Harvested cells were lysed in a radioimmunoprecipitation assay (RIPA) lysis buffer (sc-24948, Santa Cruz Biotechnology, Inc.) for Western blot analysis.

### 4.5. Enzyme-Linked Immunosorbent Assays

Enzyme-linked immunosorbent assay (ELISA) kits for human ADAMTS-1 (Cat No DY2197) and ADAMTS-4 (Cat No DY4307) were purchased from R&D Systems. Levels of human ADAMTS-1 and ADAMTS-4 in culture medium were determined according to the manufacturer’s instructions.

### 4.6. Western Blot Analysis of Human Vitreous Fluid, Human Retinal Müller Glial Cell and Human Retinal Microvascular Endothelial Cell Lysates and Rat Retinas

Retina and cell lysates were homogenized in Western blot lysis buffer (30 mM Tris-HCl; pH 7.5, 5 mM EDTA, 1% Triton X-100, 250 mM sucrose, 1 mM sodium vanadate, and a complete protease inhibitor cocktail from Roche (Mannheim, Germany)). After the centrifugation of the homogenates (14,000× *g* for 15 min, 4 °C), protein concentrations were measured in the supernatants (DC protein assay kit; Bio-Rad Laboratories, Hercules, CA, USA). Equal amounts (30–50 μg) of the protein extracts from lysates were subjected to SDS–PAGE and transferred onto nitrocellulose membranes. To determine the presence of ADAMTS proteinases, MMP-15 and the degradation products of the proteoglycan substrates versican and biglycan in the vitreous samples, equal volumes (15 μL) of vitreous samples were boiled in Laemmli’s sample buffer (1:1, *v*/*v*) under reducing conditions for 10 min.

Immunodetection was performed with the use of a mouse monoclonal anti-versican antibody (1:1000; sc-47769, Santa Cruz Biotechnology Inc.), mouse monoclonal anti-biglycan antibody (1:1000; sc-100857, Santa Cruz Biotechnology Inc.), mouse monoclonal anti-ADAMTS-1 antibody (1:1000; sc-47727, Santa Cruz Biotechnology Inc.), rabbit polyclonal anti-ADAMTS-2 antibody (1:1000; ab125226, Abcam), rabbit polyclonal anti-ADAMTS-4 antibody (1:1000, ab185722, Abcam), rabbit polyclonal anti-ADAMTS-5 antibody (1:1000, ab41037, Abcam), rabbit polyclonal anti-ADAMTS-13 antibody (1:1000, ab28274, Abcam) and mouse monoclonal anti-MMP-15 antibody (1:1000; sc-80213, Santa Cruz Biotechnology Inc.). Nonspecific binding sites on the nitrocellulose membranes were blocked (1.5 h, room temperature) with 5% non-fat milk made in Tris-buffered saline containing 0.1% Tween-20 (TBS-T). Three TBS-T washings (5 min each) were performed before the secondary antibody treatment at room temperature for 1 h. The secondary antibodies included goat anti-rabbit immunoglobulin (SC-2004) and goat anti-mouse immunoglobulin (SC-2005) (1:2000, Santa Cruz Biotechnology Inc.). To verify equal loading, membranes were stripped and reprobed with β-actin-specific antibody (1:3000, sc-47778, Santa Cruz Biotechnology Inc.). Bands were visualized with the use of high-performance chemiluminescence (G: Box Chemi-XX8 from Syngene, Synoptic Ltd., Cambridge, UK), and the band intensities were quantified with the use of GeneTools software (Syngene by Synoptic Ltd., Cambridge, UK).

### 4.7. Statistical Analysis

Data were collected, stored and managed in a spreadsheet using Microsoft Excel 2010 software. Data were analyzed and figures prepared using SPSS version 21.0 (IBM Inc., Chicago, IL, USA). A Shapiro–Wilk test and Q–Q plots were used for normality testing. Consequently, the normally distributed data were presented as mean ±SD (standard deviation) and range; otherwise, the data were presented as median (IQR) (interquartile range), and box-and-whisker plots. One-way ANOVA and an independent t-test or Kruskal–Wallis and Mann–Whitney tests (applying Bonferroni correction wherein necessary) were used to test the differences between the groups for normally distributed data and non-normally distributed data, respectively. Pearson’s correlation coefficients were used to correlate the angiogenic activity of PDR epiretinal fibrovascular membranes with the numbers of stromal-cell-expressing ADAMTS proteinases and MMP-15. Any output with a *p* below 0.05 was interpreted as an indicator of statistical significance.

## 5. Conclusions

In conclusion, we observed the upregulated expression of ADAMTS-1, -2, -4, -5 and -13 and the proteoglycans versican and biglycan cleavage products in the ocular microenvironment of patients with PDR. Because ADAMTS inhibitors have been shown to prevent inflammation and tissue destruction in animal models of arthritis [44], our findings suggest that ADAMTS proteinases, particularly, ADAMTS-4 may be key players of inflammatory cell infiltration and vascular wall destruction in PDR and possible targets for therapy. However, because of delicate balances and possible interactions between these proteinases, the significance of ADAMTS proteinases in PDR angiogenesis remains to be elucidated and deserves further investigation. Additional studies in animal models are needed to determine the specific roles of the ADAMTS proteinases in the initiation and progression of PDR and whether these proteinases represent a valuable therapeutic target for the treatment of diabetic retinopathy.

## Figures and Tables

**Table 1:** Mean numbers of immunoreactive blood vessels and stromal cells in epiretinal fibrovascular membranes from patients with proliferative diabetic retinopathy.

	Immunoreactive Blood Vessels Mean ± SD (Range)	Immunoreactive Stromal Cells Mean ± SD (Range)
ADAMTS-1	47.3 ± 20.6 (19–80)	78.6 ± 29.4 (27–145)
ADAMTS-2	61.6 ± 29.4 (21–115)	114.9 ± 55.1 (20–230)
ADAMTS-4	62.3 ± 25.5 (25–120)	146.5 ± 49.8 (62–270)
ADAMTS-5	38.3 ± 16.8 (11–70)	86.1 ± 34.9 (33–145)
ADAMTS-13	2.6 ± 9.4 (0–34)	28.5 ± 21 (10–65)

## Data Availability

Data are available from the authors upon request.
